# Association of cytokines with endothelium dependent flow mediated vasodilation (FMD) of systemic arteries in patients with non-ischemic cardiomyopathy

**DOI:** 10.1186/1476-7120-5-49

**Published:** 2007-12-10

**Authors:** Katja B Vallbracht-Israng, Ilkay Kazak, Peter L Schwimmbeck

**Affiliations:** 1 Department of Cardiology, Campus Virchow Hospital, Charité Medical University, Augustenburger Platz 1, 13353 Berlin, Germany; 2 Department of ENT, Campus Benjamin Franklin, Charité Medical University, Hindenburgdamm 30, 12200 Berlin, Germany; 3 Department of Cardiology, Hospital Leverkusen, Am Gesundheitspark 11, 51375 Leverkusen, Germany

## Abstract

**Background:**

Aim of this study was to elucidate the relation between localised inflammatory heart disease and endothelial dysfunction in the peripheral circulation, considering circulating cytokines as a potential link.

**Methods:**

In 38 patients with non-ischemic heart disease, myocardial biopsies were examined for myocardial inflammation (immunohistology) and virus persistence (PCR). Cytokines (sIL-4, IFN-g, IFN-b, IFN-a, sIL-12p7, TNF-a) were measured by ELISA in venous serum. Endothelial function of the radial artery was examined by ultrasound, measuring diameter changes in response to reactive hyperemia (FMD), compared to glyceroltrinitrate (GTN-MD). Patients with EF < 35% were excluded.

**Results:**

Age 44 ± 14 years, 19 male, 19 female, EF 63.5[16]%. FMD 4.38 [4.82]%. 30 patients had myocardial inflammation (8 not), 23 virus persistence (15 not). FMD correlated significantly with sIL-12p7 (p = 0.024, r = -0.365), but not with other cytokines. sIL-12p7 levels were significantly higher in patients with severely impaired FMD (n = 17), compared with normal FMD (n = 21): 10.70 [10.72] vs. 4.33 [7.81] pg/ml (p = 0.002). Endothelium independent vasodilation (GTN-MD 23.67 [8.21]%) was not impaired.

**Conclusion:**

Endothelial dysfunction of peripheral arteries in patients with non-ischemic cardiomyopathy is associated with elevated serum concentrations of sIL-12p7, but not of other cytokines. Circulating sIL-12p7 may partly explain, that endothelial dysfunction is not restricted to the coronary circulation, but involves systemic arteries.

## Background

Endothelial dysfunction in patients with non-ischemic heart disease, which has been observed in the coronary circulation [1] as well as in systemic arteries [2,3], is associated with myocardial inflammatory immune responses. Other groups have demonstrated endothelial dysfunction in systemic infections, like after thyphoid vaccination [4], Kawasaki disease [5], in systemic vasculitis [6] and in association with elevated CRP-levels [7]. In the Framingham Offspring Study with 2701 patients, an association between systemic inflammation and endothelial dysfunction was confirmed [8]. Endothelial dysfunction may determine prognosis, as has been demonstrated for patients with atherosclerosis [9-11] and after transplantation [12]. Non-invasive measurement of endothelial dysfunction is preferred to invasive measurements. However, the link between peripheral endothelial dysfunction and non-ischemic heart disease, needs to be determined. Inflammatory parameters have been associated with an increased risk of cardiovascular events [13,14] or the progression of heart failure [15], similar to the observations for endothelial function. Therefore, we consider circulating cytokines a potential link between non-ischemic heart disease and peripheral endothelial dysfunction. Heart failure itself is associated with endothelial dysfunction [16-19] as well as with changes in the pattern of circulating cytokines [20,21], however endothelial dysfunction of peripheral arteries is also observed in patients with non-ischemic heart disease and only mildly or regionally impaired left ventricular function. The aim of this study was to elucidate the relation between non-ischemic heart disease (in the absence of heart failure) and endothelial dysfunction in the peripheral circulation, considering circulating cytokines potential candidates for a link.

## Methods

We included 38 patients with non-ischemic heart disease, considering history, physical examination and non-invasive tests, that were referred to our hospital for acquisition and examination of myocardial biopsies. Inclusion criteria were 1. suspected cardiomyopathy by history and symptoms (chest pain, dyspnea, palpitations, exercise intolerance) or by history and ECG-changes (ST-/T-deviations, rhythm disturbances) and 2. echocardiographic regional wall motion abnormalities or global left ventricular dysfunction. Duration of symptoms was at least 3 months. By left ventricular catheterization and angiography, coronary artery disease was excluded, left ventricular ejection fraction and pressures were measured. Right-ventricular catheterization was performed to obtain endomyocardial biopsies and perform hemodynamic measurements. To minimize other confounding factors on endothelial dysfunction, patients with coronary artery disease [9-11], diabetes [22], more than one other risk factor for arteriosclerosis [22-24], overt arteriosclerosis or other severe disease were excluded from this study. As heart failure is known to affect endothelial function [16-19] as well as cytokine levels [20,21], we excluded patients with an ejection fraction <35%. At the time of the study, the majority of patients was already on cardiovascular medication [19], which was ceased according to half-life prior to the study, though this may not be necessary [25]. Sinus rhythm was required. The patients did not receive any immunomodulatory therapies.

Informed consent was obtained from all patients.

### Endothelial function

Endothelial function of the radial artery was measured, as published previously [2,3]. By high resolution ultrasound, diameter changes in response to reactive hyperemia (FMD), compared to glyceroltrinitrate (GTN-MD), were detected, referring to the standard protocols [26-28]. Flow mediated vasodilation in response to reactive hyperemia (FMD) represents endothelium dependent vasoreactivity, whereas vasodilation in response to glyceroltrinitrate (GTN-MD) indicates endothelium independent smooth muscle cell function.

#### Calculations

FMD represents the percentage of diameter increase caused by shear stress, compared to baseline. GTN-MD represents the percentage of diameter increase induced by GTN, compared to baseline.

### Cytokine measurements

Venous blood samples were obtained from the cubital vein from each patient by standard venous puncture. Samples were immediately centrifuged and the serum stored at -80°C. Cytokine measurements (sIL-4, IFN-g, IFN-b, IFN-a, sIL-12p7 and TNF-a) were performed with standard sandwich enzyme immunoassay (ELISA) kits according to the manufacturer's instructions: Hölzel Diagnostica Handels GmbH Pelikine Compact TM human IFN ELISA kits (sensitivity 1pg/ml), Hölzel Diagnostica Handels GmbH Pelikine Compact TM human IL-4 ELISA kit (sensitivity 0.2 pg/ml), Genzyme Diagnostics PredictaHuman Total Interleukin 12 ELISA kit (sensitivity 10 pg/ml).

### Myocardial biopsies

Endomyocardial biopsies from the right ventricular septum were obtained by standard percutaneous transvenous femoral approach with a standard bioptome.

#### Immunohistology

For immunohistological evaluation, the samples were prepared and evaluated as published previously [1-3,29,30]. Immunohistologically stained leucocytes (CD2+, CD3+, CD4+, CD8+ and activated CD45RO+ lymphocytes, macrophages) were counted per high-power field (400-fold magnification, equivalent to 0.28 mm^2^). Endothelial expression of HLA-1, HLA-DR and ICAM-1 was scaled (1,2,3), according to intensity of immunoperoxidase staining. Myocardial inflammation was confirmed in myocardial biopsies, if more than 7 CD3+ lymphocytes per 1 mm^2 ^tissue were identified and/or if endothelial expression of cell adhesion molecules was enhanced. Myocardial biopsies were examined and analyzed by two independent blinded observers.

### Statistical analysis

Statistical analysis was performed with the SPSS Inc. (Chicago, Illinois) software, version 12.0 for IBM-PC. Descriptive data are expressed as median and interquartile range. Quantitative data were compared to qualitative data by use of the Kruskal-Wallis test on rank sums for independent samples, followed by a post hoc multiple comparison procedure, if appropriate. To compare quantitative data of two groups, the Mann Whitney U test was applied. Quantitative data were correlated by use of the Spearman *p *rank-order analysis, calculating the coefficient of correlation r. Statistical significance was inferred at p < 0.05.

## Results

### Patients characteristics

#### General characteristics

Mean age of the 38 patients was 44.5518 years, 19 were male, 19 female. At the time of investigation, all patients were normotensive (9 treated for hypertension) and had normal lipid-levels (8 treated with statins), 4 were smokers. Of the patients on standard cardiovascular medication, 11 were treated with ACE-inhibitors, 1 with AT1-antagonists, 9 with betablockers, 2 with calciumantagonists and 5 with digitalis glucosides.

#### Clinical presentation and history

29 patients presented with chest pain (angina), 12 with palpitations, 34 with fatigue or exercise intolerance. At the time of inclusion in the study, all patients were in NYHA-stage I–II.

#### Non-invasive examinations

ST-segment or T-wave changes were documented in the ECGs of 11 patients, rhythm disturbances in 9 patients. Echocardiography revealed regional wall motion abnormalities or an impaired global systolic left ventricular function, according to the inclusion criteria. Pericardial effusions were not observed.

#### Hemodynamic measurements

Left ventricular ejection fraction (EF) was 63.5[16]%, left ventricular enddiastolic pressure (LVEDP) 74 mmHg, pulmonary capillary wedge pressure (PCWP) 66 mmHg; cardiac output (CO) 5.652 l/min and cardiac index (CI) 31 (l/min/m^2^).

### Myocardial biopsies

Myocardial inflammation or endothelial activation was confirmed by immunohistology in myocardial biopsies in 30/38 patients, according to the criteria described above. In 8/38 patients no inflammatory immune response was detected. In 23/38 patients, myocardial virus persistence was demonstrated in myocardial biopsies, 15/38 had no myocardial virus persistence. Endothelial activation (sumscore) was 64, leukocyte counts were 0.8 [1.2] CD2+, 0.8 [1.1] CD3+, 0.3 [0.5] CD4+, 0.3 [0.4] CD8+, 0.8 [1.3] CD45RO and 0.95 [1.2] macrophages. Myocyte necrosis were not observed in this study population.

### Cytokine measurements

Serum concentration for sIL-4 was 37.65 [39.57], for IFN-g 4.75 [4.5], for IFN-b 34.90 [25.4], for IFN-a 1.55 [1.57], for sIL-12p7 8.31 [9.64] and for TNF-a 7.09 [11.67] pg/ml.

Serum concentrations of TNFa correlated significantly with myocardial CD3+ lymphocytes (r = -0.382, p = 0.020), no correlations were observed between TNFa and other myocardial leukocytes or other cytokines and myocardial leukocytes. No correlation was observed between cytokine concentrations and endothelial activation. Concentrations of IFNg correlated significantly with PCWP (p = 0.001, r = 0.571), no correlations were observed between the other cytokines and hemodynamic parameters. Concentrations of IFNb and IFNa were inversely correlated (p = 0.041, r = -0.333), no correlations were observed between the other cytokines.

CRP-levels were below 6 mg/l and white cell counts were normal in the study population.

### Endothelial function

#### General Characteristics

Heart rate and blood-pressure (systolic and diastolic) did not change significantly during measurements with reactive hyperemia and after application of glyceroltrinitrate. Adequate reactive hyperemia was achieved in all subjects. Mean resting diameter of the radial artery was 2.34[1] mm at baseline, after reactive hyperemia the mean diameter increased to 2.52[1] mm and after glyceroltrinitrate (GTN) to 2.93[1] mm.

#### Flow mediated vasodilation

**Figure 1:**
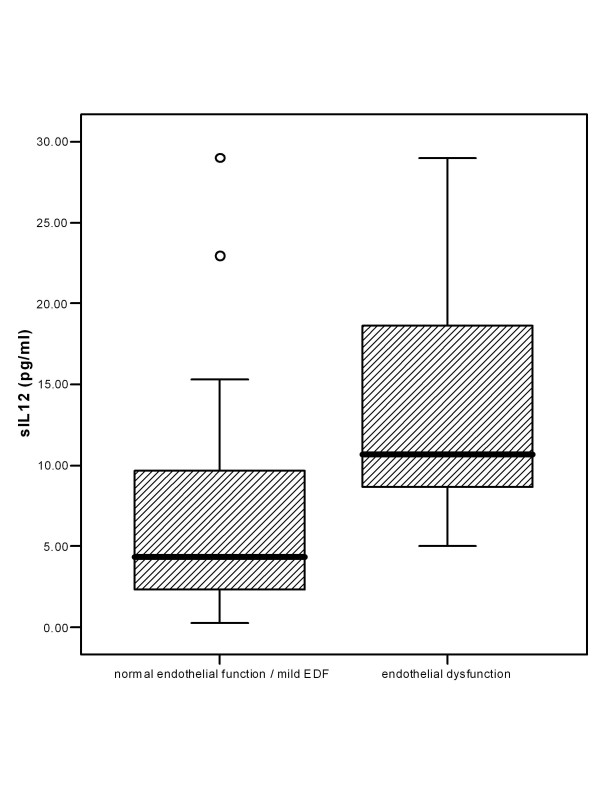
sIL-12p7 concentrations for patients with severely impaired endothelial function (FMD < 4.00, n = 17), compared with normal or moderately impaired endothelial function (FMD >= 4, n = 21) (p = 0.002).

**Figure 2:**
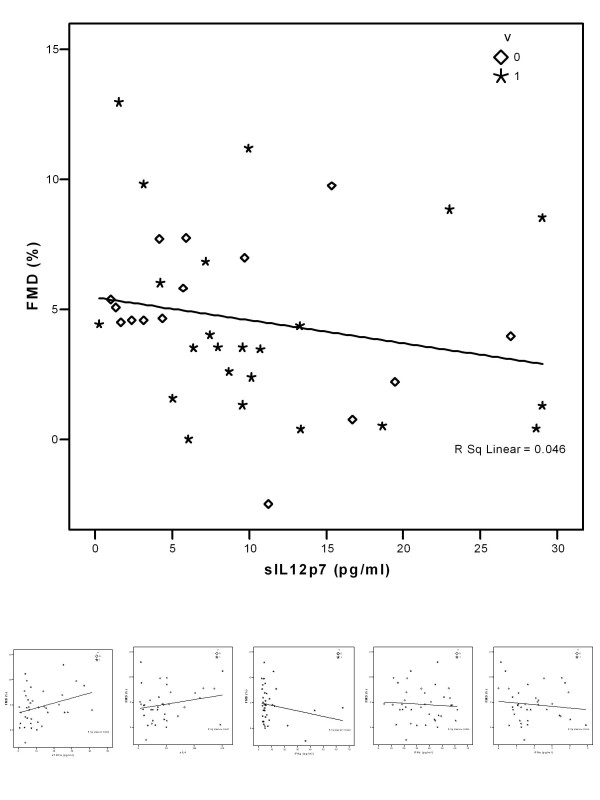
Endothelial function (FMD) of peripheral arteries in relation to circulating cytokine concentrations: sIL12 (top), TNF-a, sIL4, IFNg, IFNb and IFNa (bottom). Significant correlation for FMD and sIL12 (p = 0.024), but not for other cytokines.

Endothelial function, as determined by FMD of the radial artery, was impaired in the study population (FMD 4.38 [4.82]%). sIL-12p7 levels were significantly higher in patients with severely impaired FMD (FMD < 4.00, n = 17), compared with normal or moderately impaired FMD (FMD >= 4, n = 21): 10.70 [10.72] versus 4.33 [7.81] pg/ml (p = 0.002) (Fig. 1). FMD correlated significantly with serum concentrations of sIL-12p7 (p = 0.024, r = -0.365), but not with sIL-4 (p = 0.360, r = 0.153), IFN-g (p = 0.275, r = -0.182), IFN-b (p = 0.498, r = -0.113), IFN-a (p = 0.594, r = -0.089) and TNFa (p = 0.167, r = -0.229) (Fig. 2). FMD correlated significantly with myocardial CD3+ leukocytes (p = 0.015, r = -0.398) and CD4+ leukocytes (p = 0.023, r = -0.374).

In the patient subgroup with myocardial inflammation, similar correlations were observed as for the total study population, additionally however, we found a significant correlation between FMD and TNFa (p = 0.017, r = 0.432).

#### Endothelium independent vasodilation

Endothelium independent vasodilation in response to GTN was not significantly impaired in the study population (GTN-MD 23.67 [8.21]%). No significant correlations were observed between GTN-MD and sIL-4, IFN-g, IFN-b, IFN-a, sIL-12p7 and TNF-a. GTN-MD correlated significantly with myocardial CD3+ (p = 0.024, r = -0.370) and CD45RO leukocytes (p = 0.002, r = -0.487).

#### Impact of age and hemodynamic parameters

Age, ejection fraction and other hemodynamic measurements did not vary extensively among the study population, therefor, therse parameters had no significant impact on endothelial function or cytokine concentration or endothelial activation, with the exception of a correlation between PCWP and IFNg (p = 0.001, r = 0.571).

## Discussion

Endothelial dysfunction of peripheral arteries has been associated with myocardial inflammation and myocardial virus persistence, even in the absence of severely impaired left ventricular function [1-3]. The link between peripheral endothelial dysfunction and non-ischemic myocardial disease has remained obscure until now. In this study, we have demonstrated for the first time a relationship between circulating cytokines and peripheral endothelial dysfunction in patients with non-ischemic myocardial disease with preserved or only mildly impaired left ventricular function. Generalized inflammatory immune responses are known to be associated with peripheral endothelial dysfunction [4-8]. In this study, we focused on patients with chronic myocardial disease, so far thought off to be localized, where significant acute systemic inflammation or acute myocarditis was excluded.

We found, that endothelial dysfunction of peripheral arteries in patients with non-ischemic heart disease is associated with elevated sIL-12p7 concentrations in venous serum. sIL-12p7 activates NK-cells and displays proinflammatory actions which may potentially result in endothelial dysfunction. For sIL-4, IFN-g, IFN-b and IFN-a no association with endothelial dysfunction was observed. For s-IL-4 this was expected, as it inhibits cytokine and protease production and therefor may protect endothelium. IFN-g can display proinflammatory as well as antiinflammatory properties, which may explain, that a correlation with endothelial function was observed for sIL-12p7 but not for IFN-g.

In patients with myocardial inflammation, there was also a significant correlation between TNFa and endothelial function, impaired FMD is associated with lower TNFa concentrations. TNFa activates macrophages, induces expression of cellular adhesion molecules, and enhances prostaglandine and metalloproteinase production. It is elevated in patients with heart failure [20,21], but studies with the intention to block its actions have not conclusively shown prognostic benefits. In our study population with only mild or regional LV-dysfunction, TNFa levels were not markedly elevated. Reduced TNFa concentrations in the presence of myocardial inflammation may reflect an impaired ability to induce an adequate immune response, with damage of the endothelium by the inflammation inducing agent.

The association between endothelial function and circulating cytokines in patients with non-ischemic cardiomyopathy helps to understand pathomechanisms in myocardial disease and may explain symptoms. Whether cytokine production is localized to areas of inflammation or virus persistence in the heart or if it is due to generalized vascular inflammation cannot be differentiated by this study. Endothelial dysfunction may be induced by circulating cytokines through decreased production or decreased bioavailability of endothelium derived nitric oxide.

## Conclusion

Elevated cytokine sIL-12p7 concentrations in the peripheral circulation in patients with non-ischemic heart disease are associated with systemic endothelial dysfunction. These circulating cytokines may explain, that in patients with non-ischemic heart disease endothelial dysfunction is not restricted to the coronary circulation, but also involves systemic arteries. This finding is clinically important, as endothelial function represents an important predictor of prognosis [9-12] and may influence therapeutic decisions.

## Conflict of interests

The author(s) declare that they have no competing interests.
